# Inferior vena cava leiomyosarcoma: preoperative diagnosis and surgical management

**DOI:** 10.1186/s40792-015-0036-2

**Published:** 2015-04-10

**Authors:** Karla Elizabeth Moncayo, Juan José Vidal-Insua, Ana Troncoso, Raúl García

**Affiliations:** Complejo Hospitalario Universitario de Pontevedra, Mourente s/n, Pontevedra, 36071 Spain

**Keywords:** Inferior vena cava, Leiomyosarcoma, IVC tumor

## Abstract

Inferior vena cava (IVC) leiomyosarcoma is a rare malignant neoplasm more commonly seen in women in the fifth to sixth decade of life. Complete resection of the tumor with negative margins is the only therapeutical option that has demonstrated a survival benefit. This report presents a case of a 67-year-old woman affected by a lower segment IVC leiomyosarcoma incidentally detected during a chronic abdominal pain study. The patient was treated with tumorectomy, resection and ligation of the infrarenal IVC without signs or recurrence on a 12-month follow-up.

## Background

Soft tissue retroperitoneal leiomyosarcomas are rare malignant tumors that account for 0.5% of all sarcomas in adults and carry a poor outcome [1-3]. The vascular system is the most commonly affected arising from the smooth muscle cells of the inferior vena cava (IVC) in half of the cases [1,4,5]. It is more common in women in the fifth to sixth decade of life [1]. Diagnosis may be delayed with presenting features ranging from asymptomatic, due to their deep origin, to non-specific symptoms, due to compression, that may include palpable abdominal mass, abdominal pain, lower limb edema, thrombosis, and venous stasis [1,6]. Most information about treatment comes from case reports or small series, and complete resection of the tumor with negative margins is the only therapeutical option that has demonstrated a survival benefit [3,7-9].

We present a case of a lower segment IVC leiomyosarcoma incidentally detected during a chronic abdominal pain study. Authors and the patient as well consent to publish the information below about this case report.

## Case presentation

**Figure 1:**
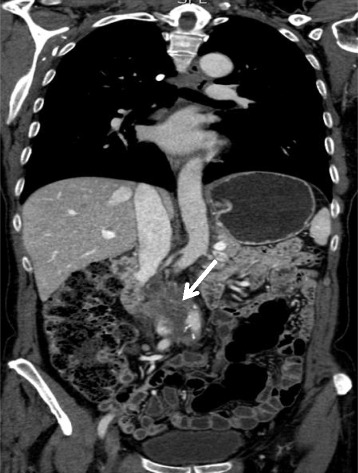
**CT scan image.** Coronal view showing retroperitoneal heterogeneous soft tissue density mass that compresses and thrombosis the infrarenal IVC of 8 × 4.6 cm in size (white arrow).

**Figure 2:**
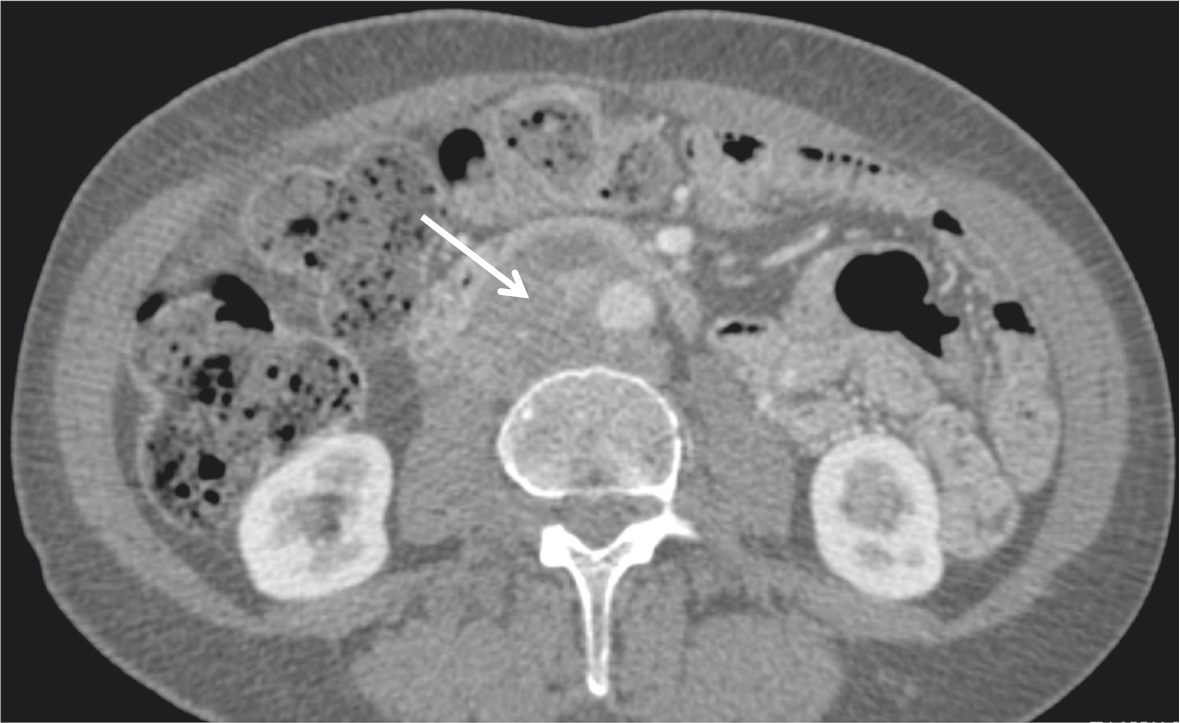
**CT scan image.** Axial view showing retroperitoneal heterogeneous soft tissue density mass that compresses and thrombosis the infrarenal IVC (white arrow).

The patient is a 67-year-old woman with relevant medical history of colon cancer treated with hemicolectomy, radiation, and chemotherapy 14 years ago without recurrence during the follow-up. The patient had several visits to the emergency room for immediate postprandial abdominal pain (especially epigastric and on upper quadrants) as well as nausea, vomiting, and significant weight loss during the last 18 months. Ultrasound demonstrated thrombosis of the inferior vena cava at epigastric level with an 80 × 30 mm thrombus. Computed tomography (CT scan) revealed a retroperitoneal heterogeneous soft tissue density mass that involved the infrarenal IVC without affecting the iliac veins of 8 × 4.6 cm in size and no evidence of metastatic disease (Figures 1 and 2). Celiac and mesenteric arteries were patent without stenosis. A biopsy of the tumor wall was conducted through a median laparotomy, because of technical difficulties by laparoscopic approach due to previous abdominal surgery and pericava localization of the tumor. As there was no definitive intraoperative diagnosis and the patient was not informed about the therapeutical procedure, tumor extirpation was postponed until pathology results were available and so the patient can be fully informed, as well. Pathologic study demonstrated a fusocellular stromal tumor, showing fascicles which intersect at 90°, histochemically and immunohistochemically concordant with IVC leiomyosarcoma (LMS).

**Figure 3:**
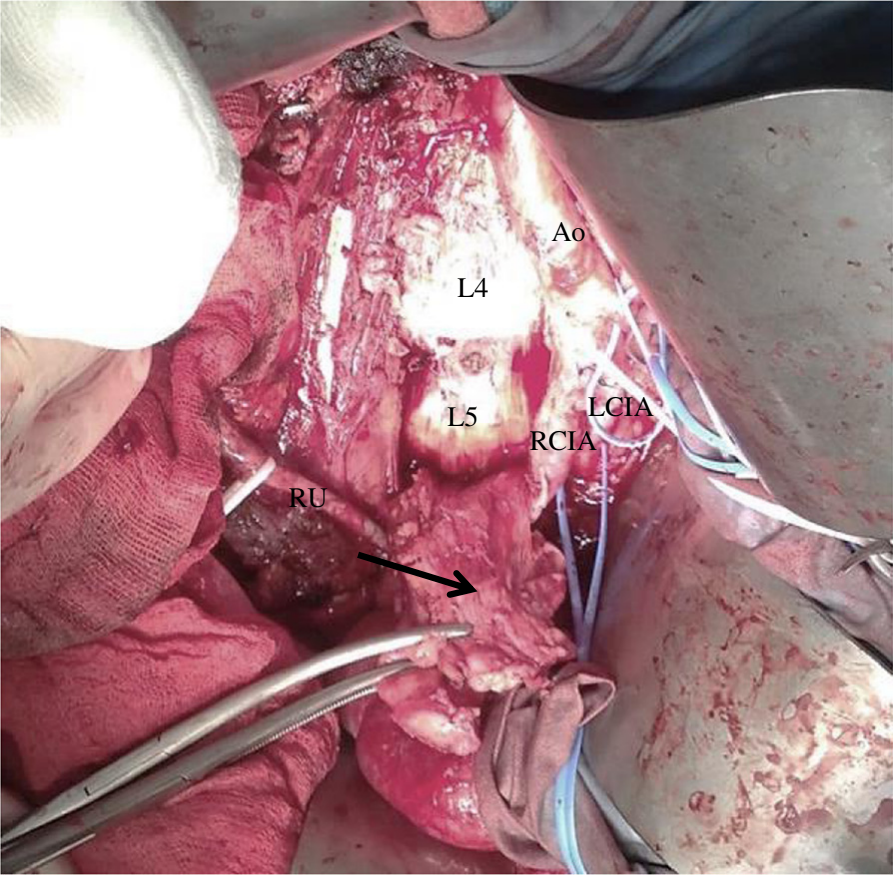
**Intraoperative image showing inferior vena cava leiomyosarcoma during tumorectomy (black arrow).** Ao, aorta; RCIA, right common iliac artery; LCIA, left common iliac artery; L4, fourth lumbar vertebra; L5, fifth lumbar vertebra; RU, right ureter.

Surgical approach by transverse abdominal incision is done, and the retroperitoneal mass arising from segment I IVC was exposed through a Kocher maneuver and right colon mobilization from the renal to the iliac veins. Inflammatory tissue and fibrosis were found and dissected carefully to preserve the surrounding structures, especially both ureters and renal veins. Tumorectomy, resection and ligation of the infrarenal IVC, was made leaving the iliac veins patent (Figure 3).

**Figure 4:**
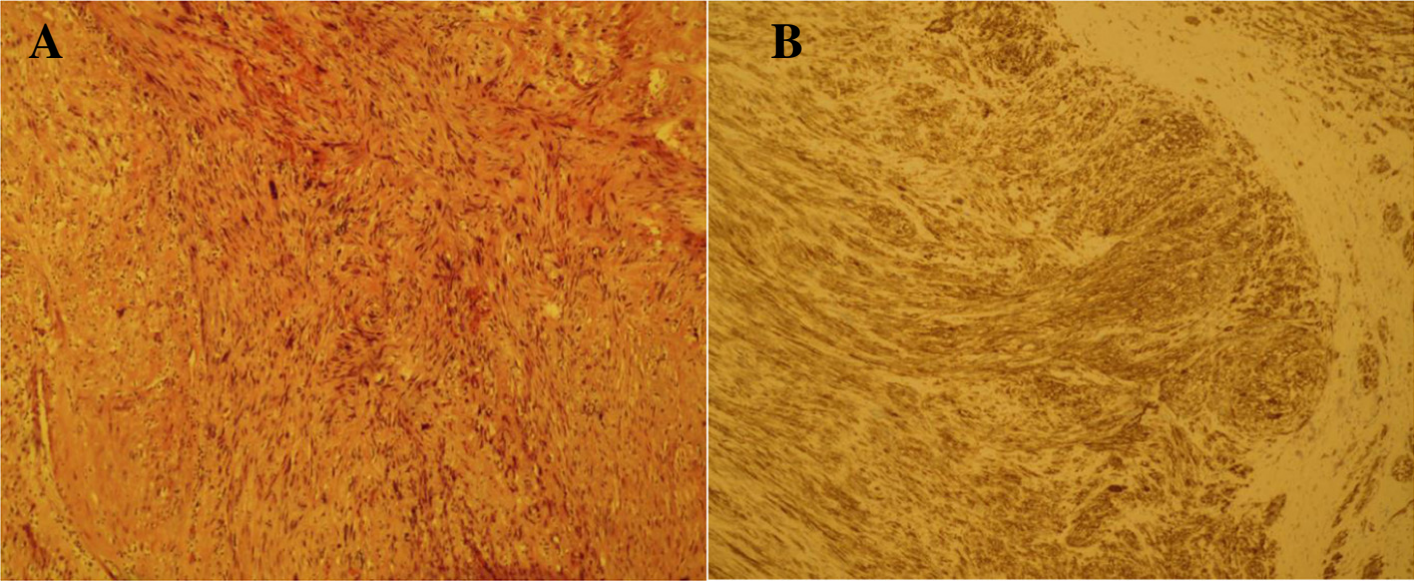
**Inferior vena cava leiomyosarcoma histological features. (A)** Microphotograph of the tumor with hematoxylin and eosin stain showing moderately differentiated IVC leiomyosarcoma, with frequent areas of hyalinization, foci of nuclear hyperchromatism and pleomorphism, and images of intraluminal growth. **(B)** Microphotograph of immunohistochemical actin stain, showing positive tumor cells with smooth muscle markers.

The specimen was composed of nine pieces, the principal of 7.5 × 3.5 × 2.5 cm in diameter, diagnosed as moderately differentiated IVC LMS, with frequent areas of hyalinization, foci of nuclear hyperchromatism and pleomorphism, and images of intraluminal growth (Figure 4A). Immunohistochemically, tumor cells were positive with smooth muscle markers (Figure 4B).

One week after the intervention, the patient developed an acute episode of hypotension, anemization, and severe abdominal wall hematoma due to spontaneous bleeding from epigastric collateral vessels probably secondary to anticoagulation therapy, which required reintervention for drainage and hemostasis. Postoperatively, mild lower limb edema was observed without signs of phlegmasia that improved with compression stockings. Afterwards, the clinical outcome was satisfactory, and upon discharge, the patient was anticoagulated first with low molecular weight heparin and then changed to acenocumarol with a target INR between 2 and 3.

At 12-month follow-up, the patient has no abdominal pain, and the CT shows that there are no radiologic signs of recurrence. Meanwhile, she is on a careful follow-up program by the oncology department, and no adjuvant therapy has been performed.

## Discussion

Malignant soft tissue tumors (STT) account for <1% of malignant tumors in adults. LMS make up less than 5% of these STT, and specifically vascular LMS accounts 0.7% of all STT [10]. Two percent of LMS originates from the vascular system being IVC, the most commonly involved [1]. Retroperitoneal sarcomas are uncommon, constituting 10% to 15% of all soft tissue sarcomas (STS) with an average annual incidence of approximately 2.7 cases per million population [11]. The mean age of presentation is 53 years with a female:male ratio of 3:1 [12].

IVC LMS usually has a delayed clinical presentation due to slow growth and retroperitoneal location with intraluminal and/or extraluminal extension and spreading by local expansion along adjacent low-resistance structures [1,6]. Metastases are initially spread hematogenously and subsequently through lymph nodes and can involve the liver, lung, and/or bones [13]. Anatomically, IVC is divided in three segments, and according to the level affected by the tumor, the symptoms may vary. Segment I involvement (infrarenal IVC) usually presents with pedal edema and abdominal distention. Segment II (middle segment of IVC, from renal to hepatic veins) with nephrotic and/or Budd-Chiari syndrome. Segment III (above hepatic veins) with pulmonary embolism [1,6,9].

Retroperitoneal LMS is more often diagnosed on CT or MRI. CT scan is the imaging modality of choice for evaluation of the tumor [14]. Coronal and sagittal slices of contrast-enhanced CT can reliably detect the origin and extension of it or if metastases are present, helping the preoperative surgical planning and determining the prognosis. Hypodense retroperitoneal mass involving the IVC with heterogeneous post contrast enhancement is the most common imaging presentation. Hence, an IVC LMS should be suspected whenever it is seen as heterogeneously enhancing as a retroperitoneal mass along the IVC [6,14]. Radiographic findings that indicate unresectability are peritoneal implants, distant metastases, involvement of the root of the mesentery, and spinal cord involvement [15]. In our case, we decided to confirm the diagnosis with biopsy of the tumor in order to be sure that it was not a recurrence of her previous colon cancer.

The only curative treatment that has been proved for IVC LMS is complete resection of the tumor with free margins. Adjuvant radiation and chemotherapy remains controversial and unclear [2,4,7,12,16,17]. Surgical options include partial resection and primary cavoplasty, complete resection and graft placement, and ligation of IVC. The latter can be performed uneventfully if a good collateral network has been developed before IVC occlusion or if it has not affected segment III IVC as it happened in our patient [3,7-9].

It has been described by Ferrario et al. on a study of 130 patients with retroperitoneal STS that local recurrence rate after local excision is 63% and after wide excision 39% (*P* < .02). The estimated 5- to 10-year survival with local recurrence was 54% and 39%, respectively, and without local recurrence 66% and 58%, respectively (*P* < .05). The overall estimated 5-year survival from the first surgery was 60% and 10-year survival 48%. Prognostic factors for survival were the grade (*P* < .001) followed by the procedure, wide versus local excision (*P* < .01), whereas tumor size was not significant [18]. According to Italiano et al. on a study of 1,472 patients with sarcoma, vascular origin is an independent adverse prognostic factor for metastasis-free survival and overall survival [19]. Meanwhile, Laskin et al. on a study of 40 patients with IVC LMS demonstrated that level III IVC and right atrial involvement by sarcoma, intraluminal growth, compromised liver, and residual postsurgical macroscopic disease negatively impact on clinical course of the disease [12].

## Conclusions

An enhanced retroperitoneal heterogeneous mass along the IVC should be a warning, and consideration of diagnosis of IVC LMS and an aggressive resection of it should be made as it is the only treatment that has proven survival benefits.

## Consent

Written informed consent was obtained from the patient for publication of this case report and any accompanying images. A copy of the written consent is available for review by the Editor-in-Chief of this journal.

## Abbreviations

CT: computed tomography
IVC: inferior vena cava
LMS: leiomyosarcoma
MRI: magnetic resonance image
